# Confidence and the Stock Market: An Agent-Based Approach

**DOI:** 10.1371/journal.pone.0083488

**Published:** 2014-01-08

**Authors:** Mario A. Bertella, Felipe R. Pires, Ling Feng, Harry Eugene Stanley

**Affiliations:** 1 Department of Economics, Sao Paulo State University (UNESP), Sao Paulo, Brazil; 2 Center for Polymer Studies and Department of Physics, Boston University, Boston, Massachusetts, United States of America; 3 Companhia do Metropolitano de Sao Paulo,Sao Paulo, Brazil; 4 Department of Physics and Centre for Computational Science and Engineering, National University of Singapore, Singapore, Singapore; University of Maribor, Slovenia

## Abstract

Using a behavioral finance approach we study the impact of behavioral bias. We construct an artificial market consisting of fundamentalists and chartists to model the decision-making process of various agents. The agents differ in their strategies for evaluating stock prices, and exhibit differing memory lengths and confidence levels. When we increase the heterogeneity of the strategies used by the agents, in particular the memory lengths, we observe excess volatility and kurtosis, in agreement with real market fluctuations—indicating that agents in real-world financial markets exhibit widely differing memory lengths. We incorporate the behavioral traits of adaptive confidence and observe a positive correlation between average confidence and return rate, indicating that market sentiment is an important driver in price fluctuations. The introduction of market confidence increases price volatility, reflecting the negative effect of irrationality in market behavior.

## Introduction

The efficient market hypothesis (EMH), defined by Fama [1] and established as the central proposition of traditional finance theory, asserts that prices consistently reflect all the information available to market traders. According to the EMH, investors earn above-average returns in financial markets by exposing themselves to greater risk. Thus individuals interacting in financial markets are assumed to be fully rational and to be maximizers of the expected utility of their wealth. Although this simplification of individual behavior has become central in the field of finance, it cannot explain several important properties of financial markets, e.g., long memory and fat tails [2]–[4].

A number of studies have indicated that investors acting in financial markets exhibit behavior that deviates from the rational behavior assumed by the traditional EMH. There are several empirical anomalies observed in financial markets that challenge the EMH approach to finance, but these can be explained by using a behavioral finance approach to examine the behavioral biases present in the decision-making process of investors.

In contrast, psychologists and scientists have documented that investors interacting in financial markets do not behave in accordance with the EMH assumption of rational behavior, but instead systematically violate the principles of expected utility, Bayesian learning, and rational expectations. Lux et al. [5] propose that herding patterns partially explain agent behavior, and a similar mechanism is proposed by Cont et al. [6]. Duffy et al. [7] propose that social learning among agents was also a factor. Gabaix et al. [8] assume that trade splitting behaviors among investors also affect market dynamics. Sato et al. [9] propose a dealer model based on past prices to help explain the statistical behavior of price fluctuations.

The recent development of tools utilizing computational modeling and artificial intelligence has allowed us to create computational simulation models that are based on the interaction of autonomous agents with distinct behavioral features. Among the most important of these are agent-based modeling techniques [10]. Their use enables us to explore the heterogeneous behavior of economic agents in financial markets and to explain some of the empirical market behavior that contradicts the EMH, e.g., bubbles, speculative movements, financial crisis, excess volatility of asset prices, and fluctuations in trading volume.

The purpose of this paper is to present an agent-based model that uses a behavioral finance approach in which the agents exhibit a behavioral bias in their decision-making process (their confidence changes in response to their degree of ongoing success, or lack thereof, in the stock market). Using this recently-developed analytical methodology we are able to examine how this behavioral bias impacts financial markets. Note that there are very few studies that incorporate psychological characteristics into agent-based models, among them the work of Takahashi and Terano [11] and Lovric [12]. We contribute to this effort by examining how confidence levels affect agent behavior and thus stock price fluctuations. We construct a model based on the Santa Fe artificial market, but modify it by allowing the agents to form their expectations based on pre-set rules and distinguishing between fundamentalist and chartist behavior patterns. The approaches taken by the above-cited papers differ from ours—Takahashi and Terano [11] base their work on a Bayes correction model and Lovric [12] base theirs on the Levy, Levy, and Solomon [13] model.

The paper is organized as follows. Section I describes the agent-based model framework. Section II explains how the expectations of the agents are determined. Section III gives additional details concerning the implementation of the model. Section IV describes the behavioral bias possessed by agents in their decision-making process. The results of the computational simulations conducted will then be presented. These simulations allow the analysis of aggregate financial market behavior when the interaction of heterogeneous agents with a given behavioral bias is examined. The last section presents some final considerations.

## Methods

### Model Framework

Before we can simulate the interaction of heterogeneous agents in financial markets, we must create an artificial stock market. LeBaron [14] describes how building an agent-based artificial financial market involves a number of design problems associated with the trading environment. In the trading environment of our artificial stock market 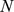 agents decide between two investment options, (i) a risky asset, i.e., a stock divided into 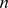 units that pays a stochastic dividend 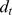, or (ii) a free-risk security that pays a constant interest rate 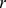 and that has an infinitely elastic supply. Time is discrete and is indexed by 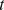, and the time horizon is set according to the experiments conducted. The dividend 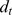 paid by the stock at each time period is generated by an exogenous stochastic process, identical with that described by Arthur et al. [15] and LeBaron et al. [16], a first order autoregressive process AR(1),
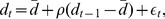
(1)where 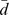 is the base dividend, 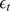 has a normal distribution with mean zero and finite variance 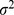, and 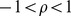. The agents have identical constant absolute risk aversion (CARA) and a utility function of wealth, i.e.,
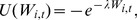
(2)where 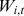 is the wealth of agent 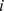 at time 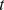, and 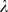 is the risk aversion level. Each agent 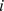 has the same initial wealth value 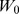. For the other time periods, the value of total wealth of agent 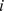 at subsequent time 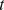 is determined to be
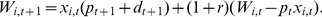
(3)where 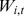 represents the wealth of agent 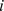 at time 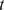, 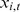 represents the number of stocks sought by agent 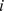, 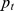 and 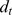 are respectively the price and dividend of the stock at time 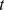, and 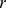 corresponds to the interest rate of the risk-free asset constant over time.

In this model, each agent tries to optimize their respective allocation of risky assets and risk-free assets. The task facing each agent at each time period is maximizing the expected utility of their wealth,
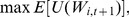
(4)subject to the constraint given by Eq. (3). Taking into consideration the utility function of wealth defined in (2), and assuming that the price and dividend expectations of the agents for a stock over the next time period are normally distributed with mean 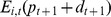 and variance 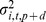, the expected utility of wealth function deriving from this utility function can be written in terms of the mean and variance of the possible results. Hence, according to [17],
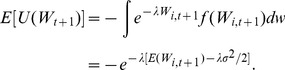
(5)


According to the maximization problem, the number of stocks demanded by agent 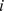 defined as 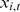 is
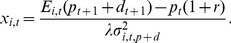
(6)


The number of stocks demanded is thus proportional to the difference between the agents' price and dividend expectations for the next period of time and the current price corrected by interest rate 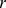, and inversely proportional to the measure of absolute risk aversion 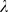 and the perceived variance of returns 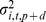.

The perceived variance of returns, 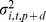, is
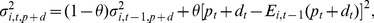
(7)where parameter 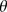 determines the weight placed on the most recent square error as opposed to the weight placed on past square errors. This parameter is of primary importance, for the more weight agents give to recent deviations, the more their behavior will become noisy and their trading more volatile.

After determining the optimum number of stocks demanded by agent 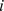 at each time period, the dynamics for determining the market price, described by Chen and Yeh [18] and Farmer and Joshi [19], is as follows. Designating 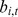 to be the number of stocks agent 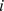 wants to buy at time 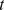, and 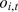 the number of stocks agent 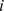 wants to sell at time 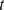, we find that
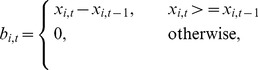
(8)and
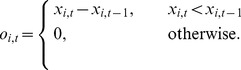
(9)


Hence
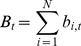
(10)and
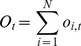
(11)are the demand and supply totals respectively.

Thus the market price of a stock at time 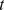 is determined through a price adjustment in terms of a surplus demand of stocks. According to Farmer and Joshi [19], a market impact function is here derived to adjust the stock price. The format of this function allows the market price to be always positive, i.e.,
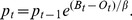
(12)


Here 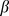 represents a scale factor that normalizes the surplus demand in the stock market and thus minimizes market fluctuations. The rate of return on stocks in the artificial financial market consists of two elements:

- Capital Gains: The stock price is collectively determined by all investors through the interaction between the total supply and demand in the market.
- Dividends: Distributed by a company at each time period according to Eq. (1) above.

Hence,
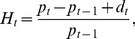
(13)where 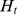 is the overall rate of return for the stock at time 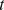.

### Formation of Expectations and Trading Strategies

Agent-based models allow us to use a range of methods when determining the expectations and trading strategies used by different groups of agents. This capability in agent-based models is the most distinguishing feature. Note that, because many agent-based models allow the trading strategies adopted by agents to evolve and improve with the use of such tools of artificial intelligence as genetic algorithms, fuzzy logic, and neural networks, the behavior of the agents becomes increasingly realistic.

In this paper the expectations concerning future stock prices and possible dividends held by agents are formed according to fixed, predetermined rules. Four rule categories are set, each of which can be adopted by agents to form their future price and dividend expectations for a market-traded stock—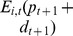. Initially the agents can be characterized as either fundamentalists or chartists. The chartists can then be sub-divided into three groups based on the memory length they use to determine their expectations. Note that the interaction among groups of agents with differing behavioral rules affects the aggregate behavior of the market. The rules for expectation formation that agents can adopt will be discussed in greater detail below.

### Fundamentalists

Fundamentalist agents estimate the future value of the stock by using the future discounted dividend flow model (the Gordon model). In this trading strategy the risky asset value forecast is based on its fundamental value, the dividend paid by the stock. The agents note the value of a stock dividend paid in the current period and, based on this value, assume the stock dividend will grow at a constant rate,
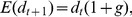
(14)where 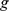 is the dividend growth rate. Using the future discounted dividend flow model, the expected future price of a stock is defined to be
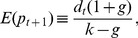
(15)where 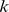 refers to the discount rate of the future dividend flow. Using these equations we determine the value of 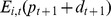, which is then used to determine the optimum number of stocks to be purchased by each agent 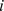 at each time period.

### Chartists

Recent trends in the literature show an increasing interest in chartist trade behavior, sometimes called “noise trading” [20], [21]. Chartist agents forecast the future price and dividend of a risky asset by assuming that price changes are affected by inertia, i.e., if the stock price has recently increased, it will continue to increase, and if it has decreased, it will continue to decrease. Reference [22] defines chartists as those who keep track of past average prices in order to be either trend followers or trend contrarians. Takahashi and Terano [11] distinguish between three types of chartist agents, categorizing them according to the length of their memory when they analyze the price history of a stock and make a forecast. Empirical findings supporting this assumption [23]–[25] have demonstrated that agent memories are indeed heterogeneous.

The expectation of a future stock price is defined to be
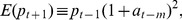
(16)and the expectation of a future stock dividend is defined to be
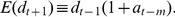
(17)


The forecast rules are then categorized according to memory length 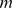,

- when 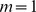, 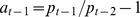,
- when 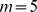, 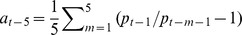, and
- when 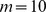, 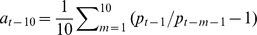.

Note that this moving average mechanism is similar to mechanisms proposed by other researchers [11], [22] for defining the strategies of chartists, with some variation in the details of the calculations used. Using this definition of chartists, in Sec. V we will introduce the confidence factor as we analyze chartist behavior and how market sentiment affects market dynamics.

### Model Implementation

After determining the main elements constituting the artificial financial market, the computational simulations can be carried out. The artificial financial market is implemented using LSD (Laboratory for Simulation Development) software, a platform written in C++ for the development, use, and distribution of computational simulations. This software is suitable for the implementation of agent-based models because it creates simulations in discrete time, and results are expressed as a series of values for each variable of the model. The computational simulations are executed according to the following steps.

1. At the beginning of each time period 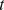, the value of dividend 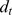 is generated.
2. The agents then make their predictions in terms of stock price and dividend for the next time period 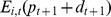. The agents can be fundamentalists or chartists, depending on the rules they use for their predictions.
3. After the expectations of the future price and dividend of the stock are defined, the number of stocks demanded by the agents at time period 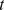 [Eq. (6)] is set.
4. Using Eqs. (8) and (9), the buy and sell stock orders by the agents are determined.
5. The buy and sell stock orders are added to the market.
6. Using Eq. (12), the market price of the stock is then adjusted to reflect the surplus stock demand in the market.
7. After the market price of the stock for time period 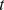 is defined, the agents' asset portfolio and the wealth level for the current time period are updated. Equation (7) for the perceived variance of returns is also updated for use in the next time period. The information on both the aggregate behavior of the market and the individual behavior of the agents is recorded for later analysis.

In all the simulations, the artificial market consists of 100 agents and each run is for 5,000 time steps. Each agent is allowed only five stocks during each time period. Short selling of up to five stocks is permitted. These restrictions are kept uniform in artificial financial markets so that replication of the results is more realistic. Because chartists must look at past 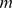 periods to make their decisions, in the first 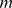 period we assume the behavior of the chartists will match that of the fundamentalists.

### Overconfidence and Self-attribution Bias

Barberis and Thaler [26] divide studies of behavioral finance into two categories, (i) those attempting to show that arbitrage transactions in financial markets are not perfect, i.e., are not always effective in allowing asset prices to remain connected to their fundamental values, and (ii) those attempting to use a study of the psychology of decision-makers to demonstrate that agents make systematic errors because of uncertainty, i.e., they deviate from neoclassical assumptions in terms of maximization of utility, stable preferences, and optimal information processing.

Overconfidence is considered a judgment bias related to the cognitive psychology of the decision-maker and has received a great deal of attention in financial studies. According to Kahneman and Riepe [27], because financial decisions are made in environments that are highly complex and uncertain, agents rely on fixed decision-making rules and intuition. When intuition is given excessive weight, overconfidence can affect investment decisions and agents end up encountering unknown risks, experiencing unanticipated outcomes, and engaging in reckless trading.

Studies of the psychology of agents strongly indicate that traders are overly confident in their ability to predict the future and overestimate the accuracy of their data. Experimental evidence presented by Aldrighi and Milanez [28] indicates that in a sampling in which agents were instructed to point out the variation ranges of some variable with a confidence level of 90%, they were able to indicate the ranges including the correct value only 70% of the time. The study presented by Barberis and Thaler [26] documents that agents tend to overestimate or underestimate the probability attribution, i.e., events they believe will occur with 100% probability in fact occur in only 80% of the cases, and events they believe cannot occur in fact occur in 20% of the cases.

Odean [29] proposes that there are reasons to expect that agents actively trading in financial markets will be more confident in their investment abilities than the population in general. Investors who have been successful in the past may over-evaluate the degree to which they were responsible for their positive outcomes and thus become overconfident. Agents can thus have unrealistic expectations about their ability to generate future profits from market transactions and execute trades in which the expected profits are insufficient to cover trading costs. They can also overestimate the accuracy of their information or believe the information they have is relevant when in fact it is not. In this study we treat overconfidence as a calibration error and model it as an underestimation of the stock return variance. This adjustment of confidence can be understood as an adaptive process in line with the so-called adaptive market hypothesis (AMH) introduced by Lo [30].

In the model, given the perceived stock return variance described by Eq. (7), a confidence coefficient is then created which, when multiplied by the perceived variance of the returns, characterizes its overestimate,
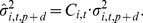
(18)


Here coefficient 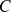 is the coefficient of agent confidence level adjustment. When 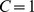, the agent has a neutral level of confidence and the variance of stock returns is not underestimated. When 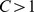, the agent has little confidence and the variance of stock returns is overestimated. When 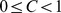, the agent is overconfident and the variance of stock returns is underestimated, i.e., the agent's prediction of the expected return of the stock is overconfident.

We assume that an agent's level of confidence evolves during the time span of the simulation. According to Odean [29], the overconfidence of extremely successful agents can further increase by the “self-attribution bias,” i.e., they believe that their success in trading is solely the result of their own abilities.

The rules for updating the confidence levels are
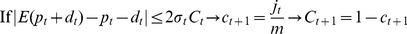
(19)

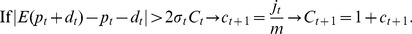
(20)


Here 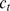 is the confidence index, 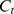 is the coefficient of adjustment of confidence, and 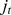 is the number of correct forecasts.

In other words, if the difference between the prices and dividends is less or equal to 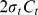, then the confidence for the next period increases and the coefficient of adjustment of confidence decreases. This coefficient decreases to reduce the perceived standard deviation of the agent. Should the difference between the expected return of the stock and the actual return fall within the confidence interval set by the agent, the confidence level is increased and multiplied by coefficient 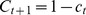, if it does not, the confidence level is decreased and the standard deviation is corrected by coefficient 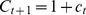.

## Results and Discussion

Table 1 shows the values attributed to the parameters of the model. We specify the initial values on the basis of configurations exhibited in several artificial financial markets, among them those created by Arthur et al. [15], Lovric [12], and Farmer and Joshi [19]. We keep the same initial parameter values in all of these simulations. The selected parameter ranges are summarized in table 2.

**Table 1:** Values attributed to general parameters.

Parameters	Values
Number of Agents	100
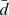	4
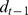	4
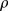	0.95
mean 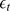	0
var 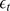	0.0742
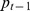	20
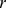	0.10
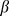	2000
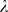	0.5
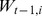	100
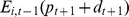	22
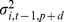	4
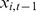	1
g	0.015
k	0.25
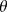	0.01

**Table 2:** Testing of the model on different parameter sets.

Usable range	Justification
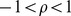	Ensure stationarity of time series.
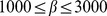	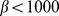 and 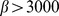 show high kurtosis in relation to returns and prices when agents are confident.
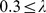	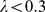 reveals very high kurtosis for returns for different agents with and without confidence.
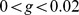	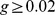 reveals high kurtosis for returns when agents are fundamentalists (100%).
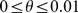	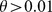 shows very high kurtosis for returns when agents are fundamentalists (100%).

We carry out the simulations as follows:

1. In the first simulation, all agents are fundamentalists.
2. In the second simulation, we take the presence of chartist agents in the market into consideration (with 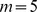) and progressively increase their participation by 25 percentage points.
3. In the third simulation, we take the behavioral heterogeneity of agents into consideration. The market is now composed of 25 fundamentalist agents, 25 chartist agents with 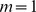, 25 chartist agents with 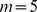, and 25 chartist agents with 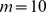.
4. The fourth simulation adds the factor of agent overconfidence to the configuration produced by (c).

### Neutral Confidence

In the first simulation all the agents are fundamentalists and the same rule for the formation of expectations—the discounted dividend flow model—is applied to all agents. We use this simulation as a reference case for comparison with the outcomes of the subsequent simulations. The agents are homogeneous and the stock price in terms of its fundamental value is the risky asset.

**Figure 1:**
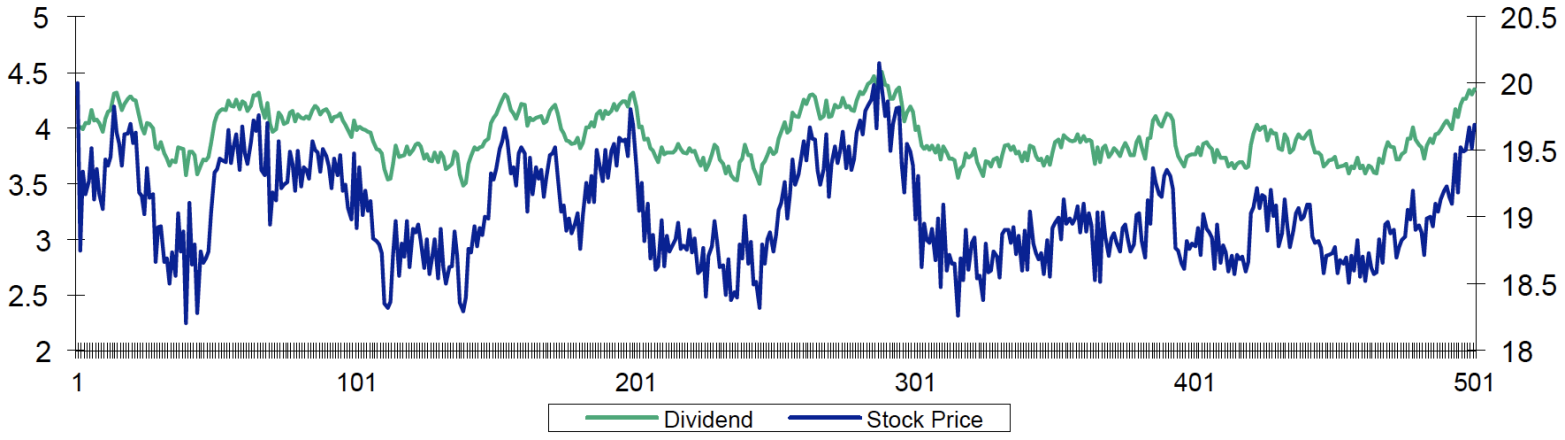
Evolution of the Dividend and Price of the Stock (Agents 100% Fundamentalist).

Figure 1 shows the evolution of dividend value and stock price. Note that the financial series obtained in this simulation has the fundamental value of the risky asset as a reference. All agents have the same information set and interpret it identically. Because the main information signal is the dividend paid by the stock, the behavior of the financial market is affected by this variable.

The EMH states that the fundamental value of asset prices fully reflect all the information available to market agents. Asset prices are thus random, i.e., price changes are unpredictable, unaffected by price history, and are impacted soley by exogenous new information made available to traders. Our results support the EMH only when all agents are assumed to be homogeneous, when they all base their market expectations solely on the fundamental value of the asset traded. When this is the case, changes in the fundamental value of the risky asset strongly affect the asset trading price.

Table 3 shows the statistics of this simulation. The frequency distribution of the stock rate of return is close to a normal distribution. The frequency is highest in the center and symmetrically decreases toward the tails. The statistics presented in this table confirm this feature, i.e., the mean and median of the return rate of stock are approximately equal and the asymmetry coefficient approaches zero. The coefficient of kurtosis indicates that the flattening of the frequency distribution is slightly larger than a normal distribution. This fact reveals the existence of more kurtosis in the stock return rate distribution than in a normal distribution.

**Table 3:** Descriptive Statistics (Agents 100% Fundamentalist).

	Dividend	Stock Price	Rate of Return
Mean	3.989391968	19.1530035	0.208230248
Median	3.99124	19.1331	0.208019
Standard deviation	0.234956843	0.430676299	0.018671856
Sampling Variance	0.055204718	0.185482075	0.000348638
Excess Kurtosis	−0.12709383	0.059072402	1.091727473
Skewness	0.042000304	0.295107615	0.095830846

**Figure 2:**
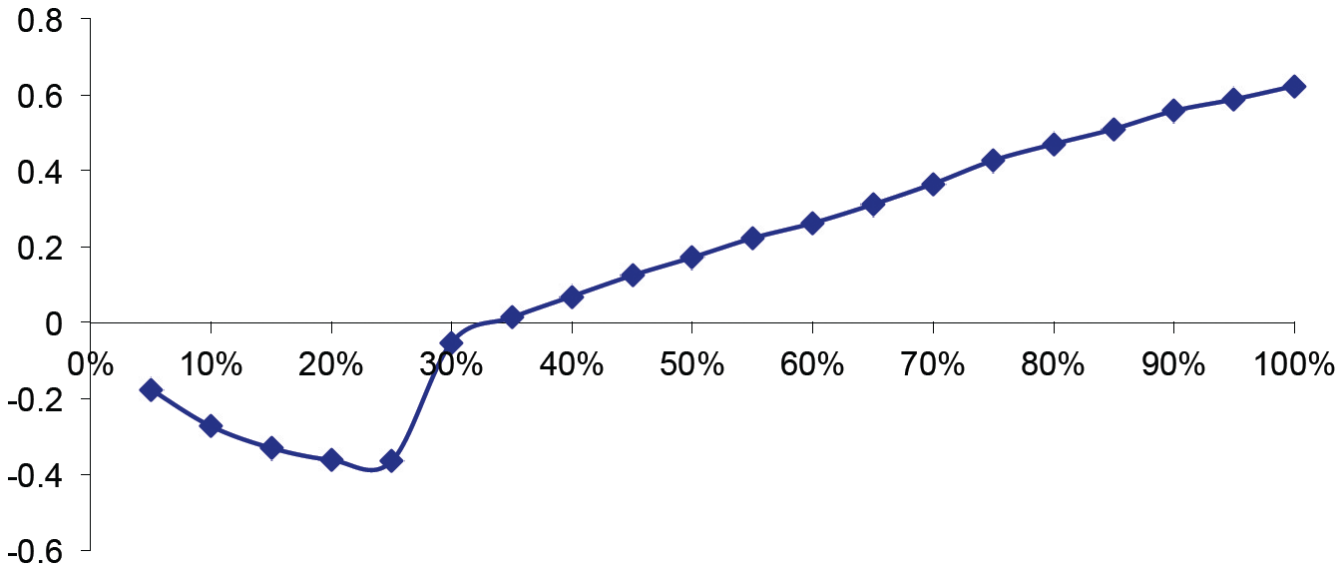
Excess Volatility of the Return Rate with increasing presence of chartists.

In the next simulation, chartists with memory length 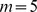 are introduced. Each subsequent realization increases the presence of chartists by five percentage points, with the rest of the agents remaining fundamentalists. Figure 2 shows the changing simulation results for excess volatility as we increase the percentage of chartists in the system.

Note that when the number of chartist agents is greater than 25% the market becomes more volatile. The greater the chartist participation, the greater the stock price fluctuations and the more extreme and periodic the fluctuations become. The impact of their actions is greater than the impact of the actions of fundamentalist agents. When the number of chartist agents decreases below 25% the market becomes less volatile, the actions of chartists have little impact, the actions of fundamentalists have greater impact, and the stock rate of return does not experience large swings. This in turn favors the predictions made by chartists. As chartist market participation then increases, market volatility increases. And thus the market alternates between periods of relative calm and periods of volatility.

**Figure 3:**
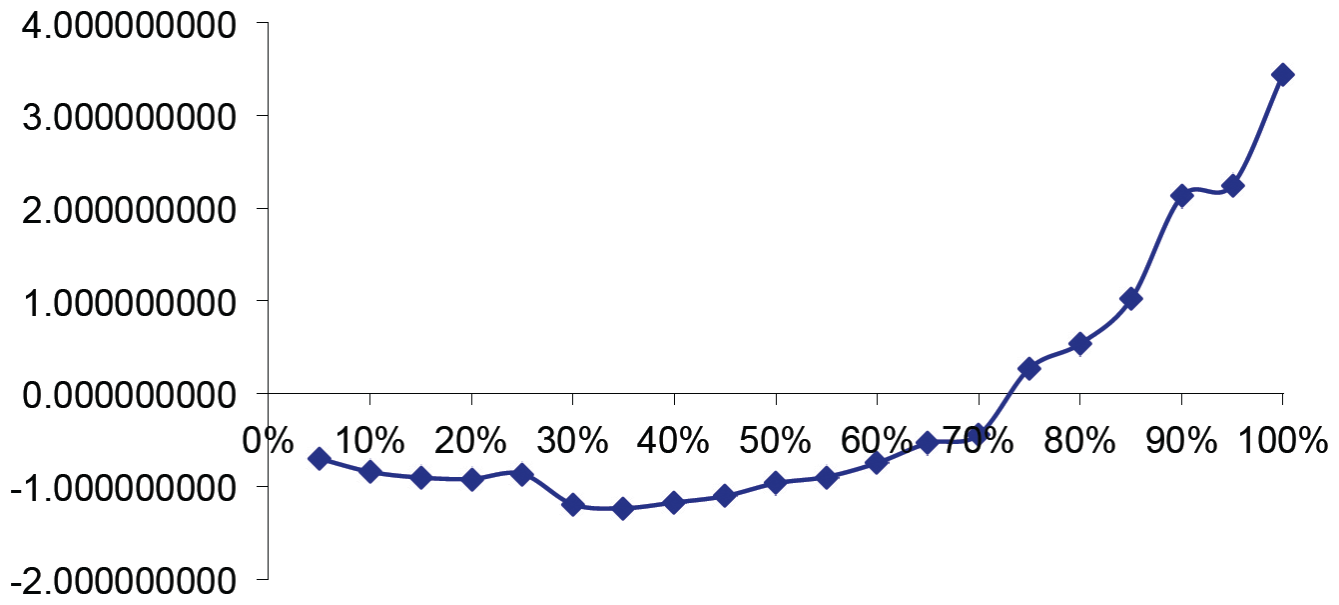
Excess Kurtosis of the Return Rate with increasing presence of chartists.

As seen in Fig. 3, the excess kurtosis significantly increases only when the percentage of chartists exceeds 75% of the total number of agents. This lends support to the finding that at least 80% of agents are chartists [31].

Table 4 shows that the degree of average value dispersion increases as the participation of chartist agents increases. The coefficient of variance and the standard deviation of the return rate of the stock confirms this fact, and both progressively increase between simulations. The weight of the distribution tails becomes increasingly heavy, i.e., with excess kurtosis, which is indicated by the kurtosis coefficients.

**Table 4:** Descriptive Statistics of the Return Rate of the Stock (Chartists 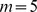)

	25%	50%	75%	100%
	Chartists	Chartists	Chartists	Chartists
Mean	0.198716887	0.201945959	0.201895818	0.201971459
Standard Deviation	0.011845511	0.021880992	0.026652911	0.030414828
Sampling Variance	0.000140316	0.000478778	0.000710378	0.000925062
Kurtosis	3.147274427	3.0369499	4.38921917	7.82753461
Skewness	−0.000345276	−0.174160967	−0.614287785	−1.219774691

**Figure 4:**
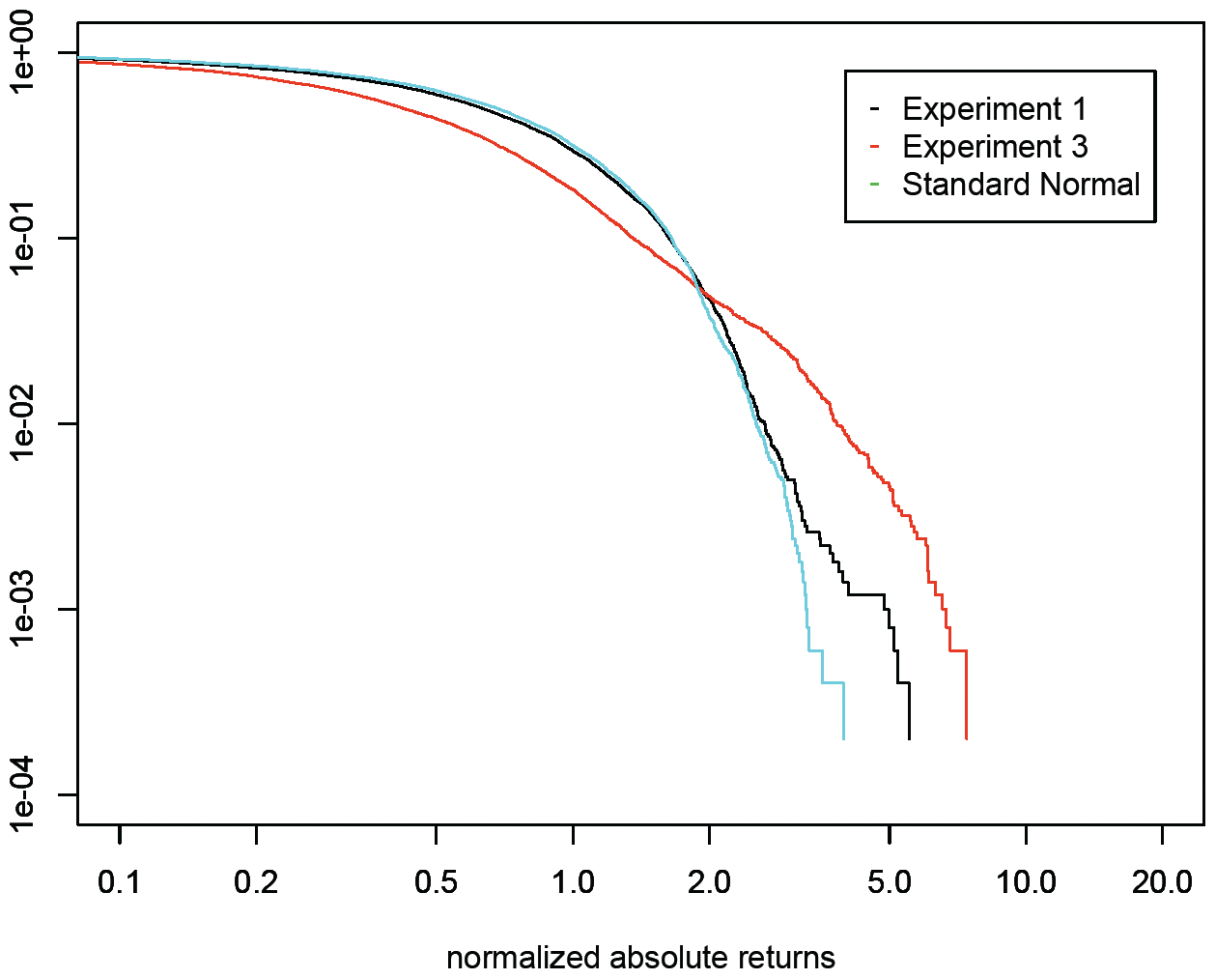
Plots of CDF of simulation result from purely fundamentalists and heterogeneous agents with chartists of different memory. While the prior exhibits a close to normal distribution, the later shows a fat tail that is more realistic.

Another simulation examines the interaction of different types of agents. Behavioral heterogeneity is modeled by allowing agents to adopt different trading strategies. The market consists of fundamentalist agents and chartist agents with differing memory lengths (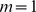, 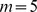, and 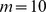). Of the total of 100 market agents, 25% are fundamentalists, 25% are chartists with memory 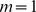, 25% are chartists with memory 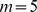, and 25% are chartists with memory 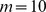. Table 5 shows the descriptive statistics produced by this simulation. Figure 4 plots the cumulative distribution function of the results from fundamentalists and from heterogeneous agents with different memory lengths. Note that the tails of the frequency distribution of the return rate become heavier and the distribution exhibits excess kurtosis, a characteristic commonly found in financial series. These characteristics are due to the behavioral heterogeneity present in the market.

**Table 5:** Descriptive Statistics (different types of agents).

	Stock Price	Rate of Return
Mean	20.56182484	0.194568287
Standard deviation	0.813071391	0.030512176
Sampling Variance	0.661085087	0.000930993
Kurtosis	8.302251611	11.811203205
Skewness	−1.369367354	0.916660916

### Overconfidence

**Figure 5:**
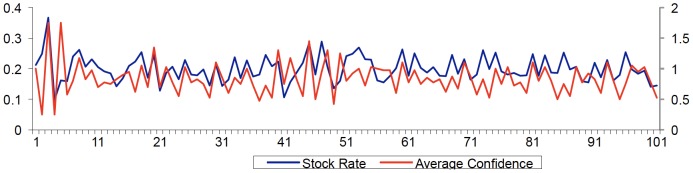
Average Confidence Level and Return Rates (Different Types of Agents with overconfidence).

We test the different configurations of our model, which allows the interaction of agents with differing trading strategies, by now assuming that agents can have an overconfidence bias. As shown above, agent overconfidence influences their estimation of the variance of stock return. This in turn influences their orders for stock purchase or sale. Therefore we focus our next simulation on the interaction between the differing types of market agent, and allow their confidence levels to evolve during the simulation time. The market is composed of 25 fundamentalists who are not influenced by confidence, and 75 chartists (divided equally according to their memory analysis) who are. Figure 5 presents the results of this simulation.

The strong presence of agents who follow market trends causes greater volatility in stock prices and return rates, features observable in real-world markets. Bubbles and crashes are also indicated as reoccurring events. Figure 5 shows that periods when return rates increase coincides with periods in which the agents are more confident. Periods when the return rate drops sharply coincides with periods in which the agents are less confident.

Table 6 shows that there is a positive correlation between the return rate (with and without dividends) and the average level of agent confidence, a result that has important implications for financial markets. Table 7 shows that the volatility measured for both stock price and return rate in terms of standard deviation increases more than in Table 5, which only takes agent heterogeneity into consideration. Including the psychological feature of confidence level increases the volatility (and the risk to assets) of stock prices and return rates. On the other hand, the kurtosis index decreases substantially, suggesting that, when rules of behavior are established, the distribution tails become less fat and approach the index of kurtosis in a normal distribution.

**Table 6:** Correlation between Average confidence and return rate.

	Average confidence
Return Rate	0.235
Return Rate w/o Dividend	0.25

**Table 7:** Descriptive Statistics (Different Types of Agents with Overconfidence).

	Stock Price	Return Rate
Mean	20.30198682	0.19733
Std. Deviation	0.822535095	0.039841
Sampling Variance	0.676563982	0.001587
Kurtosis	3.195286159	3.103466
Skewness	−0.194194136	0.014115

To compare this with real-world data, we use the price history of the S&P 500 available from Yahoo! Finance [32] and the market confidence index from the Yale School of Management [33]. Note that all the data of our model agrees well with S&P 500 data for these 13 years (see Table 8). Note also that the empirical correlation between the S&P 500 returns and the confidence index is 0.192 before August 2008 (the financial crisis) and that our simulation result is 0.25. After the crash of Lehmann Brothers in September 2008, however, the individual confidence index unexpectedly increases and provokes a small, negative correlation between the above variables (−0.043) from September of 2008 to August of 2013.

**Table 8:** Comparison of simulation result with S&P500 from Yahoo! Finance [32].

	Jan 03 2000	Dec 03 2012	Monthly return	St Deviation	Kurtosis
S&P500	1394.46	1426.19	0.014%	0.046	3.80
Simulation	20	20.7235	0.023%	0.037	3.87

### Ranges of selected parameters

To test the robustness of our results, we simulate the model on different parameter sets (see Table 2). Our major findings are valid within the broad usable range of the parameters. Outside of this usable range the model tends to exhibit excessive, unrealistic kurtosis.

## Conclusions

Behavioral finance provides a new way of analyzing financial markets. Many of the stylized facts in a financial time series contradict the central theoretical proposition in finance, i.e., the efficient market hypothesis (EMH). Empirical evidence shows that much of the behavior of individual market agents cannot be explained using conventional decision models, especially in their attitudes toward risk and their susceptibility to such biases in judgment as overconfidence.

Using agent-based modeling techniques we have examined the influence of overconfidence on the decision-making process of market agents. By applying this bias to behavioral agents, we made possible the enrichment of this recently-developed analytical methodology and demonstrated that these models can take into account additional behavioral characteristics.

By testing the interactions between market agents with differing trading strategies we are able to demonstrate that the presence of behavioral heterogeneity explains the excess volatility of risky assets relative to their fundamental value. In addition to its possible relevance to the development of new market trading strategies, this study proposes that the confidence levels of trading agents change over time and that their actions, influenced by their confidence levels, actively influence the creation of reoccurring market bubbles.

The results presented here coincide with many features found in real-world financial time series, and they contradict the results produced by traditional theories in finance. Most important, agent-based models allow us to more fully understand real-world market features than when more traditional analytical methods are used.

Note that this work represents a simple exercise of behavioral finance—using agent-based models to understand trader confidence in financial markets. Future studies could focus on risk aversion or excessive optimism or, using the tools of artificial intelligence (e.g., genetic algorithms or neural networks), examine further the changing behavioral rules followed by market agents.
